# Supplementary material for: Nutritional Strategies Prescribed During Pregnancy and Weight Gain in Women with Gestational Diabetes Mellitus: A Systematic Review of Observational Studies
Source: Nutrients. 2024 Dec 27;17(1):43. doi: 10.3390/nu17010043 (PMC11722879; doi:10.3390/nu17010043)
Supplement: Supplementary file 1 [file nutrients-17-00043-s001.zip › nutrients-3363366-supplementary.pdf]

Supplementary Materials:

Table S1: Search strategy used in the systematic review.

**Databases**	**Search Strategy**
PubMedline	(Diabetes, Gestational OR Gestational Diabetes OR Diabetes Mellitus, Gestational OR Gestational Diabetes Mellitus) AND (Diet OR Food Intake OR Dietary Intake OR Nutritional Intake OR Meals OR Diet Therapy) AND (Gestational Weight Gain OR Weight Gain, Gestational OR Pregnancy Weight Gain OR Weight Gain, Pregnancy OR Maternal Weight Gain OR Weight Gain, Maternal OR Weight Gain) AND (Cross-Sectional Studies OR Cross Sectional Studies OR Cross-Sectional Study OR Studies, Cross-Sectional OR Study, Cross-Sectional OR Case-Control Studies OR Case-Control Study OR Studies, Case-Control OR Study, Case-Control OR Cohort Studies ORCohort Study OR Studies, Cohort OR Study, Cohort)
Web of Science	Diabetes, Gestational OR Gestational Diabetes OR Diabetes Mellitus, Gestational OR Gestational Diabetes Mellitus AND Diet OR Food Intake OR Dietary Intake OR Nutritional Intake OR Meals OR Diet Therapy AND Gestational Weight Gain OR Weight Gain, Gestational OR Pregnancy Weight Gain OR Weight Gain, Pregnancy OR Maternal Weight Gain OR Weight Gain, Maternal Weight Gain AND Cross-Sectional Studies OR Cross Sectional Studies OR Cross-Sectional Study OR Studies, Cross-Sectional OR Study, Cross-Sectional OR Case-Control Studies OR Case-Control Study OR Studies, Case-Control OR Study, Case-Control OR Cohort Studies OR Cohort Study OR Studies, Cohort OR Study, Cohort<br>Case-Control Studies OR Case-Control Study OR Studies, Case-Control OR Study, Case-Control Cohort Studies OR Cohort Study OR Studies, Cohort OR Study, Cohort
Scopus	"Diabetes, Gestational" OR "Gestational Diabetes" OR "Diabetes Mellitus, Gestational" OR "Gestational Diabetes Mellitus" AND "Diet" OR "Food Intake" OR "Dietary Intake" OR "Nutritional Intake" OR "Meals" OR "Diet Therapy" AND "Gestational Weight Gain" OR "Weight Gain, Gestational" OR "Pregnancy Weight Gain" OR "Weight Gain, Pregnancy" OR "Maternal Weight Gain" OR "Weight Gain, Maternal" OR "Weight Gain" AND "Cross-Sectional Studies" OR "Cross Sectional Studies" OR "Cross-Sectional Study" OR "Studies, Cross-Sectional" OR "Study, Cross-Sectional" OR "Case-Control Studies" OR "Case-Control Study" OR "Studies, Case-Control" OR "Study, Case-Control" OR "Cohort Studies" OR "Cohort Study" OR "Studies, Cohort" OR "Study, Cohort"
Embase	'Diabetes, Gestational' OR 'Gestational Diabetes' OR 'Diabetes Mellitus, Gestational' OR 'Gestational Diabetes

	Mellitus' AND 'Diet' OR 'Food Intake' OR 'Dietary Intake' OR 'Nutritional Intake' OR 'Meals' OR 'Diet Therapy' AND 'Gestational Weight Gain' OR 'Weight Gain, Gestational' OR 'Pregnancy Weight Gain' OR 'Weight Gain, Pregnancy' OR 'Maternal Weight Gain' OR 'Weight Gain, Maternal' OR 'Weight Gain' AND 'Cross-Sectional Studies' OR 'Cross Sectional Studies' OR 'Cross-Sectional Study' OR 'Studies, Cross-Sectional' OR 'Study, Cross-Sectional' OR 'Case-Control Studies' OR 'Case-Control Study' OR 'Studies, Case-Control' OR 'Study, Case-Control' OR 'Cohort Studies' OR 'Cohort Study' OR 'Studies, Cohort' OR 'Study, Cohort'

Table S2: Analysis of the methodological quality of cohort and case-control studies using the Newcastle-Ottawa Scale (NOS).

Studies	Items								
	Selection				Comparability	Exposure			Score
	1	2	3	4	1	1	2	3	
Morisset et al. [22]	-	*	-	-	**	*	-	*	5/8
Morisset et al. [23]	*	-	*	*	-	*	-	-	4/8
Couch et al. [24]	-	*	**	*	*	*	*	*	8/8
Most; Langer [25]	-	*	-	*	**	**	-	*	7/8
Ho et al. [27]	-	*	**	-	*	*	-	-	5/8

Table S3: Analysis of the methodological quality of a cross-sectional study using the Joanna Briggs Institute (JBI) tool.

Studie	Q1	Q2	Q3	Q4	Q5	Q6	Q7	Q8	Total	Quality
Sunjaya; Sunjaya [26]	U	Y	Y	Y	NA	NA	Y	Y	5	Moderate

Legend: Y= Yes; N= No; U= Unclear; NA= Not applicable; Q1. Were the criteria for inclusion in the sample clearly defined? Q2. Were the study subjects and the setting described in detail? Q3. Was the exposure measured in a valid and reliable way? Q4. Were objective, standard criteria used for measurement of the condition? Q5. Were confounding factors identified? Q6.

Were strategies to deal with confounding factors stated? Q7. Were the outcomes measured in a valid and reliable way? Q8. Was appropriate statistical analysis used?
